# Genetic Diversity of Multidrug-Resistant *Pseudomonas aeruginosa* Isolates Carrying *bla*_VIM–2_ and *bla*_KPC–2_ Genes That Spread on Different Genetic Environment in Colombia

**DOI:** 10.3389/fmicb.2021.663020

**Published:** 2021-08-27

**Authors:** Ana M. Rada, Elsa De La Cadena, Carlos A. Agudelo, Christian Pallares, Eliana Restrepo, Adriana Correa, María V. Villegas, Cesar Capataz

**Affiliations:** ^1^Department of Microbiology and Parasitology, Bacteria and Cancer Group, Universidad de Antioquia, Medellín, Colombia; ^2^Faculad de Ciencias de la Salud, Biociencias Group, Institución Universitaria Colegio Mayor de Antioquia, Medellín, Colombia; ^3^Grupo de Resistencia Antimicrobiana y Epidemiología Hospitalaria, Universidad El Bosque, Bogotá, Colombia; ^4^Clinica Universitaria Bolivariana, Medellín, Colombia; ^5^School of Health Sciences, Universidad Pontificia Bolivariana, Medellín, Colombia; ^6^Facultad de Ciencias Básicas, Universidad Santiago de Cali, Cali, Colombia; ^7^Fundación Clínica del Norte, Bello, Colombia

**Keywords:** *Pseudomonas aeruginosa*, genetic diversity, *bla*_KPC–2_, *bla*_VIM–2_, integron, plasmid

## Abstract

*Pseudomonas aeruginosa* is an opportunistic Gram-negative pathogen with an increase in the frequency of infections caused by multidrug resistant (MDR) and extensively drug resistant (XDR) strains, limiting the available therapeutic options. The most troublesome resistance is the acquisition and production of carbapenemases such as Verona integron-encoded metallo-β-lactamases (VIM), the most frequent and widespread, and the *Klebsiella pneumoniae* carbapenemases (KPC), which has continuously spread in the last decade. Its dissemination is linked to their location on mobile genetic elements (MGEs). In Colombia, VIM and KPC have been increasing in its frequency showing major successful dissemination. In this article, we molecularly characterized and analyzed the genetic context of *bla*_VIM_ and *bla*_KPC_ in carbapenem-resistant *P. aeruginosa* (CRPA) isolates from infected and colonized patients in two tertiary-care hospitals, one in Medellín and the other in a municipality close to Medellín, both areas with high carbapenemase endemicity in Colombia (2013–2015). Using whole-genome sequencing (WGS), we identified a remarkable variety of genetic backgrounds in these MDR *P. aeruginosa* isolates carrying *bla*_KPC–__2_ and *bla*_VIM–__2_. There were a diversity of class 1 integron and variations in the gene cassettes associated to *bla*_VIM–__2_, as well as a possible event of spread of *bla*_KPC–__2_ mediated by a plasmid that contained part of Tn*4401*b in one infection case. The dissemination of *bla*_VIM–__2_ and *bla*_KPC–__2_ in *P. aeruginosa* in this area in Colombia has been strongly influenced by successful international clones, carrying these genes and additional determinants of resistance on MGEs, accompanied by gene rearrangement under an antimicrobial selection pressure. These findings emphasize the need to implement control strategies based on rational antibiotic use.

## Introduction

*Pseudomonas aeruginosa* is an opportunistic Gram-negative pathogen especially in immunocompromised patients capable of causing a wide array of life-threatening infections. In hospitals, *P. aeruginosa* plays a crucial role in healthcare-associated infections (Pachori et al., 2019). The increasingly frequent infections caused by multidrug resistant (MDR) and extensively drug resistant (XDR) strains with limited therapeutic options are associated with high morbidity and mortality (Magiorakos et al., 2012; Horcajada et al., 2019). The intrinsic resistance is conferred by low outer membrane permeability, expression of efflux pumps, and the production of antibiotic inactivating enzymes. The acquired resistance can occur because of mutational changes or acquisition of resistance genes via horizontal transfer by mobile genetic elements (MGEs), such as integrons, transposons, or plasmids (Horcajada et al., 2019).

In particular, the most troublesome acquired resistance of *P. aeruginosa* is the production of carbapenemases, which confer resistance to most commercially available β-lactam. The class B carbapenemases, such as Verona integron-encoded metallo-β-lactamases (VIM) and Imipenem metallo-β-lactamases (IMP), are the most frequent (Patel and Bonomo, 2011). The genes encoding IMP and VIM are located on integrons, which also carry other antibiotics resistance genes favoring their worldwide dissemination (Hong et al., 2015; van der Zee et al., 2018). *P. aeruginosa* carrying class A carbapenemases such as *Klebsiella pneumoniae* carbapenemases (KPC) have become increasingly important due to their continuous dissemination worldwide including Asia and America during the last decade (Santella et al., 2012; Carrara-Marroni et al., 2015; Hu et al., 2015; Potron et al., 2015). Its dissemination is facilitated by genes encoded on transposable elements and plasmids (Naas et al., 2013; Dai et al., 2016; Hu et al., 2019). In Colombia, VIM-producing *P. aeruginosa* was first reported in 2006, followed by *P. aeruginosa* harboring KPC in 2007 (Villegas et al., 2006, 2007). The presence of these carbapenemases in high-risk clones identified by multilocus sequence typing (MLST) as sequence type (ST) 111 (ST111) harboring *bla*_VIM–__2_ on a class 1 integron such as In59 (Correa et al., 2015; Vanegas et al., 2014), as well as ST308 and ST235 harboring *bla*_KPC–__2_ on complete or truncated Tn*4401*b in plasmids or the chromosome, may be the cause of its successful dissemination in Colombia (Cuzon et al., 2011; Abril et al., 2019).

The study of genetic platforms of *bla*_VIM_ and *bla*_KPC_ in carbapenem-resistant *P. aeruginosa* (CRPA) that co-harbor genes conferring resistance to other antibiotics, as well as their wide diversity, is key to understand the role in the dissemination of such resistance determinants among clinical and environmental isolates (Cuzon et al., 2011; Naas et al., 2013; Correa et al., 2015; Abril et al., 2019). In this article, we molecularly characterized and analyzed the genetic context of *bla*_VIM_ and *bla*_KPC_ in CRPA isolates from infected and colonized patients in two tertiary-care hospitals, one in Medellín and the other in a municipality close to Medellín, both areas with high carbapenemase endemicity in Colombia (2013–2015).

## Materials and Methods

### Bacterial Isolates and Clinical Data

A collection of CRPA isolates (*n* = 46) from a surveillance study of carbapenem-resistant Gram-negative bacteria was selected from infected and colonized patients in two tertiary-care hospitals in Colombia between 2013 and 2015. Thirty-eight isolates were recovered from hospital 2 located in a municipality close to Medellin, while the remaining isolates were collected from hospital 1 located in the city of Medellin (143 and 202 beds, respectively; see Supplementary Data Sheet 1). The medical records of infected and colonized patients were reviewed retrospectively. Colonization was defined as a CRPA recovered from a surveillance rectal culture or clinical sample without associated signs or symptoms of disease. Rectal swabs were cultured on a selective chromogenic medium (chromID CARBA; bioMérieux). Infection was defined by an associated clinical syndrome of infection. Colonization and infection were confirmed by the infectious disease services and/or infection control unit. Clinical information such as age, sex, previous hospitalization, days of hospital stay before sampling, use of invasive medical devices, underlying diseases, comorbidities, and antibiotic use was collected from electronic medical records and recorded in a Microsoft Access Database. This study was approved by the Institutional Review Board (IRB) and Ethical Committee in each participating hospital.

### Identification, Antimicrobial Susceptibility Testing, and Carbapenemases Detection

Species identification and antimicrobial susceptibility testing was performed using the automated Vitek-2^TM^ system (bioMérieux Marcy-l′Étoile, France). The antimicrobial agents tested included imipenem, meropenem, doripenem, ceftazidime, cefepime, piperacillin/tazobactam, gentamicin, amikacin, and ciprofloxacin. The minimum inhibitory concentration (MIC) results were interpreted following the Clinical and Laboratory Standards Institute (CLSI) breakpoints 2017 (CLSI., 2017). All isolates classified as carbapenem-resistant were tested by PCR assay for the presence of carbapenemases encoding genes including *bla*_VIM_, *bla*_KPC,_ *bla*_NDM_, and *bla*_IMP_. The primers used for amplification, as well as PCR cycling conditions, have been described elsewhere (Yigit et al., 2001; Dallenne et al., 2010). DNA sequencing was performed on the amplification products of positive PCR, and the results were compared and aligned with reference sequence using the online BLAST database to identify specific alleles.

### Molecular Typing and Whole-Genome Sequencing

The characterization by rep-PCR/DiversiLabTM (bioMérieux Marcy-l′Étoile, France) was conducted in 21 isolates of *P. aeruginosa* carrying the *bla*_KPC_ or *bla*_VIM_ gene that complied with DNA in good quantity and quality pos-extraction to determine the genomic relatedness, using >95% similarity to be considered to be of the same rep-PCR type. A total of 16 isolates were selected for whole-genome sequencing (WGS) based on this initial characterization. Total DNA was extracted with the GeneJET Genomic DNA Purification Kit (Thermo Fisher Scientific, Waltham, MA, United States). DNA libraries were prepared using a NexteraXT^®^ DNA sample preparation kit and multiplexed with a NexteraXT index primer kit on the Illumina platform (Illumina, San Diego, CA, United States). Genomic libraries were sequenced on a MiSeq sequencer to obtain 250-bp paired-end reads using Kit v2 and v3 (Illumina). The readings were processed to eliminate low-quality bases and contamination with sequences of adapters and later assembled *de novo*. Cleaning and assembly were carried out using a CLC Genomics Workbench assembler, version 8.5. The genomes were annotated using the RAST server.^1^ The assemblies were typed on the web server of the Center for Genomic Epidemiology using the MLST 2.0 (Multilocus Sequence Typing)^2^ (Larsen et al., 2012).

### Genome Analysis

The determination of resistance elements was identified using Resfinder 2.1^3^ (Zankari et al., 2012), using an identity percentage higher than 95% and a coverage cutoff greater than 90%. The O-specific antigen analysis was performed *in silico* in the *Pseudomonas aeruginosa* serotyper (PAst) program^4^ (Thrane et al., 2016). The Tn*4401* isoforms were determined by BLASTn comparing the region surrounding each *bla*_KPC_ gene to the sequences of the Tn*4401* isoforms as described previously (Naas et al., 2012). Overlapping sequences that comprised the region surrounding *bla*_VIM_, the different integrons, and gene cassettes were manually confirmed using BLASTn and BLASTp. The identification of the integrons was investigated using INTEGRALL, the reference database of integron sequences^5^ (Moura et al., 2009). Likewise, this database was used for registry and the integron number assignment. Easyfig^6^ was used to compare and visualize the backbone of different MGEs. The BLAST Ring Image Generator (BRIG) software (Alikhan et al., 2011) was applied to align the assembled reads of some sequenced clinical isolates to one reference plasmid carrying *bla*_KPC_. The mutations in selected resistance genes (*gyrA*, *gryB*, *parE*, *parC*, *rpoB*, *pmrA*, *pmrB*, *parS*, *parR*, *mexX*, *mexY*, *mexZ*, *mexC*, *oprJ*, *nfxB*, *mexT*, *mexE*, *mexF*, *oprN*, *mexA*, *mexB*, *oprM*, *mexR*, *nalC*, *nalD*, *oprD*, *ampC*, *ampD, ampDh2, ampDh3*, and *ampR*) in all genomes annotated were determined with reference to *P*. *aeruginosa* PAO1 (accession number NC_002516.2) using a custom pipeline.

### Phylogenetic Analyses

The phylogenetic reconstruction of the isolates was carried out by detecting single nucleotide polymorphisms (SNPs) against the reference genome of *P. aeruginosa* PAO1 (accession number NC_002516.2). Also, we included the reference genome of *Pseudomonas putida* K72440 (accession number NC-002947.4) as an external group. A SNP matrix (SNP matrix) was constructed and used to reconstruct the phylogeny of the strains with RAxML (Stamatakis, 2014). We used the general time reversal (GTR) model with a GAMMA distribution and Lewis correction for the parameters to determine the best phylogenetic reconstruction by maximum likelihood. We performed 20 runs and chose the one with the best score. In addition, 100 bootstraps were made to support the reconstructions. The trees obtained were visualized by iTOL (Letunic and Bork, 2016).

### Statistical Analysis

Comparison of clinical and epidemiological data was performed between colonized and infected patients, as well as between carbapenemase-producing *P. aeruginosa* (CPPA) and non-CPPA isolates. Fisher’s exact (two tails) or chi-square test was used for qualitative variables and Wilcoxon rank sum test for continuous variables.

### Accession Numbers

The sequence data for the isolates were submitted to the NCBI GenBank database under the BioProject number PRJNA391501.

## Results

### Infection and Colonization by Carbapenem-Resistant *Pseudomonas aeruginosa*

The 46 isolates of CRPA from the two hospitals were recovered from 41 adult patients; 24 (58.5%) were infected and 17 (41.4%) were colonized, and 7 (17.1%) of them were localized in intensive care unit (ICU) at the time of sampling in the participating hospitals. Most patients were males (68.3%, *n* = 28) and older adults (median age of 63 years; interquartile range [IQR] = 49–74). The majority of the CRPA isolates from the infected patients were from soft tissue (29.2%, *n* = 7; see Supplementary Table 1). In general, the most common underlying conditions were hypertension (48.8%, *n* = 20) and diabetes mellitus (24.4%, *n* = 10). A total of 19 patients (46.3%) had previous antibiotic exposure, with carbapenems, piperacillin-tazobactam, and glycopeptides being the most frequent (12.2% for each, *n* = 5; see Supplementary Table 1). Among the isolates recovered, 50% (*n* = 23) were positive for two of the four carbapenemases-encoding genes evaluated by PCR (2 from hospital 1 and 21 from hospital 2). The genes *bla*_VIM–__2_, *bla*_KPC–__2_, and *bla*_VIM–__2_ plus *bla*_KPC–__2_ were detected in 47.8% (*n* = 11), 47.8% (*n* = 11), and 4.3% (*n* = 1) isolates, respectively. These isolates were obtained from 19 patients; 10 were infected (*n* = 3, *bla*_VIM–__2_; *n* = 7, *bla*_KPC–__2_) and 9 were colonized (*n* = 6, *bla*_VIM–__2_; *n* = 2, *bla*_KPC–__2_; *n* = 1, *bla*_VIM–__2_ plus *bla*_KPC–__2_). Two infected patients were previously colonized by VIM-producing *P. aeruginosa* (each patient had two isolates). Also, up to three KPC-producing *P. aeruginosa* isolates were collected from different sources in different days from the same infected patient (see Supplementary Data Sheet 1).

Of the total of CRPA isolates, 91.3, 87.0, and 84.8% were resistant to doripenem, meropenem, and imipenem, respectively. More than half of the isolates were resistant to piperacillin/tazobactam (73.9%), ciprofloxacin (60.8%), cefepime (58.7%), ceftazidime (56.5%), and gentamicin (56.5%). When comparing between CPPA and non-CPPA, the resistance to gentamicin was higher in CPPA than in non-CPPA. Furthermore, more than half of the isolates of both CPPA (86.9%) and non-CPPA (65.2%) were MDR, defined as non-susceptible to at least one antibiotic in three antimicrobial categories (Magiorakos et al., 2012), with amikacin/gentamicin, cefepime, ceftazidime, imipenem, meropenem, doripenem, piperacillin/tazobactam, and ciprofloxacin in CPPA (52.2%) being the most frequent resistance (see Supplementary Table 2).

### Diverse Genetic Background of Multidrug-Resistant *Pseudomonas aeruginosa* Isolates Carrying *bla*_KPC_/*bla*_VIM_

**FIGURE 1:**
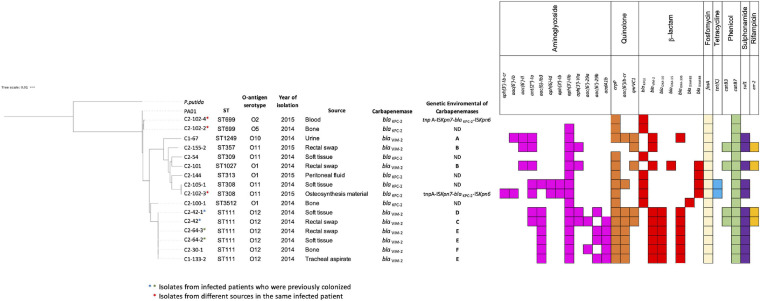
Phylogenetic tree showing the genetic relationships among isolates of *Pseudomonas aeruginosa* (*n* = 16). Isolates were characterized by ST, O-antigen serotype, and year of isolation. We indicate carbapenemases genes *bla*_KPC–__2_ or *bla*_VIM–__2_ and the genetic environment identified in each isolate. Resistance determinants to aminoglycoside, quinolone, β-lactam, fosfomycin, tetracycline, phenicol, sulfonamide, and rifampicin are grouped and indicated by color (color box indicates presence; blank indicates absence).

The initial characterization of CPPA isolates by rep-PCR/diversilab revealed five different rep-PCR-type: two of them harboring *bla*_KPC–__2_ from hospital 2 (two isolates for each) and three included isolates harboring *bla*_VIM–__2_ from hospitals 1 and 2 (two isolates for each); the other isolates were unrelated (see Supplementary Figure 1). Based on this characterization, 16 MDR *P. aeruginosa* isolates were selected for WGS (*n* = 7, *bla*_KPC–__2_; *n* = 9, *bla*_VIM–__2_). The isolates carrying the *bla*_KPC–__2_ gene (*n* = 7) recovered from infected patients exhibited a variety of genetic backgrounds, with five different ST, including the ST309 belonging to the clonal complex (CC) 309 associated with O-antigen serotype O11 (*n* = 1) and the ST308 belonging to CC308 with O11 serotype (*n* = 2); other isolates with a singleton sequence type ST313 (*n* = 1) and belonging to ST699 (*n* = 2) were associated with other serotypes. Additionally, one new ST was designated as ST3512 (*n* = 1; see Figure 1). The isolates carrying *bla*_VIM–__2_ (*n* = 9) recovered from colonized and infected patients showed four different ST profiles, with ST111 belonging to international CC111 associated with O12 serotype being the most frequent (*n* = 6), while other isolates belonged to ST1249 (*n* = 1) and ST1027 (*n* = 1), and the ST357 (*n* = 1) belonged to CC357 (Figure 1). Of note, four isolates ST111 genetically related were recovered from rectal swab samples and site of infection (soft tissue) from two patients, suggesting the colonization and infection by the same clone of *P. aeruginosa* in each patient. In general, the isolates carried other genes that can confer resistance to several antibiotics, including aminoglycosides, sulfonamides, tetracyclines, quinolones, phenicol, fosfomycin, and β-lactam (see Figure 1).

In addition, sequencing confirmed the presence of mutations in antimicrobial resistance-associated genes in some of the clinical isolates using *P. aeruginosa* PAO1 as a reference (Table 1). In general, some mutations in quinolone resistance determining regions (QRDRs) of GyrA, ParC, and ParE detected have been previously linked to fluoroquinolone resistance as well as the overexpression of efflux pump systems (Dunham et al., 2010; Subedi et al., 2018; Do Nascimento et al., 2020). In some isolates, the *mexZ*, *nfxB, mexT*, and *mexR* genes, which regulate the MexXY-OprM, MexCD-OprJ, MexEF-OprN, and MexAB-OprM multidrug efflux systems, revealed the presence of several point mutations predicted to result in several amino acid substitutions associated with resistance previously reported (Monti et al., 2013; López-Causapé et al., 2018b; Neves et al., 2019; Olsson et al., 2020). Regarding AmpC polymorphisms, the T105A substitution was detected in most of the isolates, which has been correlated with more efficient carbapenem and cefepime hydrolysis (Cabot et al., 2012). Furthermore, some mutations detected in PmrA and PmrB were observed previously in colistin-sensitive strains (Snesrud et al., 2018); also, some of the alterations in OprD had been described before with no contribution to carbapenem resistance (Horna et al., 2018; Díaz-Ríos et al., 2021).

**TABLE 1:** Mutations identified in antimicrobial resistance-associated genes of 16 sequenced isolates of *Pseudomonas aeruginosa* using PAO1 as reference.

**Gene name**	**Product**	**Alteration(s) or mutation(s) [number of isolates harboring the mutation]**	**References**	**Antibiotics affected^a^**
*gyrA*	DNA gyrase subunit A	T83I [9], G663C [1], V671I [6], S859C [1], G860S [6], D893E [6], A900G [6], and S903A [6]	*Subedi et al., 2018; Do Nascimento et al., 2020; Papagiannitsis et al., 2020	FQ
*gyrB*	DNA gyrase subunit B	wt		FQ
*parE*	DNA topoisomerase IV subunit B	E533D [8]	*Dunham et al., 2010	FQ
*parC*	DNA topoisomerase IV subunit A	S87L [9], S197L [1], and P572T [3]	*Subedi et al., 2018; Do Nascimento et al., 2020	FQ
*rpoB*	DNA-directed RNA polymerase beta chain	V51I [14]	Do Nascimento et al., 2020	RIF
*pmrA*	Two-component regulator system response regulator PmrA	L71R [11]	Snesrud et al., 2018; Do Nascimento et al., 2020	COL
*pmrB*	Two-component regulator system signal sensor kinase PmrB	S2P [10], A4T [10], V6A [6], V15I [10], G68S [10], and Y345H [14]	Snesrud et al., 2018; Do Nascimento et al., 2020	COL
*parS*	Two-component sensor	R7H [2], L137P [6], S277N [1], and H398R [16]	*López-Causapé et al., 2018a	COL, IMI, MER, CEF, AMG, FQ
*parR*	Two-component sensor	R70W [3], L153R [7], and S170N [7]	*Olsson et al., 2020	COL, IMI, MER, CEF, AMG, FQ
*mexX*	Resistance-nodulation-cell division (RND) multidrug efflux membrane fusion protein MexX precursor	A30T [4], V309I [1], K329Q [14], L331V [14], and W358R [14]	Vettoretti et al., 2009; Olsson et al., 2020	CEF, AMG, FQ
*mexY*	RND multidrug efflux transporter MexY	A501V [15], S530G [6], I536V [10]T543A [8], G589A [11], Q840E [10], N1036T [10], and Q1039R [12]	Vettoretti et al., 2009; Olsson et al., 2020	
*mexZ*	Transcriptional regulator of the mexXY multidrug transporter operon	D83E [1], G89S [2], and L138R [2]	*López-Causapé et al., 2018b	
*mexC*	RND multidrug efflux membrane fusion protein MexC precursor	R43Q [1], T142A [1], E218Q [3], A229E [3], A244T [3], H277R [6], S2806 [1], S297A [14], E314K [1], A345T [4], P350S [6], and A351V [4]		IMI, MER, FQ
*mexD*	RND multidrug efflux transporter MexD	T87S [4], A155T [1], E257Q [7], T286M [1], V434A [1], F597Y [1], V660I [1], N669D [1], S685G [1], I703V [1], S845A [14], S915A [1], I982V [1], K1031R [3], and S1040T [1]	Papp-Wallace et al., 2019	
*oprJ*	Multidrug efflux outer membrane protein OprJ precursor	D68G [2], M69V [8], A211V [1], A260V [1], Q267R [6], and T376S [1]	Papp-Wallace et al., 2019; Olsson et al., 2020	
*nfxB*	Transcriptional regulator NfxB	R21H [1], D56G [1]	*Monti et al., 2013	
*mexT*	Transcriptional regulator MexT	F94I [1]	*Neves et al., 2019	IMI, MER, FQ
*mexE*	RND multidrug efflux membrane fusion protein MexE precursor	S8F [3], A80G [1], A231T [1], D353F [2], and D370E [2]	Olsson et al., 2020	
*mexF*	RND multidrug efflux transporter MexF	D230A [2], D667E [1], and A843T [1]		
*oprN*	Multidrug efflux outer membrane protein OprN precursor	A4T [1], S13P [7], and A410S [2]	Olsson et al., 2020	
*mexA*	RND multidrug efflux membrane fusion protein MexA precursor	K76Q [1]		IMI, MER, CEF, AMG, FQ
*mexB*	RND multidrug efflux transporter MexB	I186V [1], G957D [2], S1041E [4], and V1042A [4]	Neves et al., 2019; Olsson et al., 2020	
*oprM*	Major intrinsic multiple antibiotic resistance efflux outer membrane protein OprM precursor	D448N [1]	Olsson et al., 2020	
*mexR*	Multidrug resistance operon repressor MexR	V126E [13]	*Olsson et al., 2020	
*nalC*	Transcriptional regulator NalC	G71E [14], D79E [3], A145V [2], and S209R [7]	Horna et al., 2018, 2019	
*nalD*	Transcriptional regulator NalD	W205R [1]		
*oprD*	Basic amino acid, basic peptide and imipenem outer membrane porin OprD precursor	D43N [1], S57E [4], S59R [4], T103S [3], K115T [4], V127L [9], F170L [4], E185Q [13], P186G [13], V189T [13], E202Q [10], I210A [10], E230K [10], S240T [10], N262T [10], A267S [1], T276A [9], A281G [9], K296Q [9], Q301E [9], R310E [1], G312R [6], A315G [11], L347M [8], M372V [9], N375S [9], N376S [9], V377S [9], G378S [9], K380A [9], N381G [9], Y382L [9], T3939 [6], N394T [6], L395W [6], Y399P [6], V400S [6], V401T [6], Q402S [6], S403A [3],	Díaz-Ríos et al., 2021; Horna et al., 2018	IMI, MER
		G404R [6], K407R [6], D408P [6], L409R [6], S410T [6], F411C [6], Q424E [3], and G425A [3]		
		F308_S349del [1], S373del [9], G383del [9], E396Edel [6], A397del [6], I414_L443 del [6], and W277_L443del[2]		
*ampC*	Beta-lactamase precursor	P7S [3], F19L [1], G27D [5],R79Q [1], T105A [14], Q155R [1], L200F [1]V205L [7], V356I [5], and G391A [7]	*Cabot et al., 2012; Subedi et al., 2018	C/T, CAZ/AVI
*ampD*	Beta-lactamase expression regulator AmpD	A29V [1], Q44H [1], E68D [1], G148A [9], and S175L [1]	Cabot et al., 2012; Do Nascimento et al., 2020	CAZ, CEF, PPT
*ampDh2*	Beta-lactamase expression regulator AmpDh2	V40I [2], V89D [1]		
*ampDh3*	Beta-lactamase expression regulator AmpDh3	I67T [1], A208V [1], and A219T [6]	Díaz-Ríos et al., 2021	
*ampR*	Transcriptional regulator AmpR	E114A [2], S179T [1], A194S [1], I251V [1], G283E [13], E287G [7], M288R [6], M288Q [7], A290V [7], V291L [7], and A293S [7]	Cabot et al., 2012; Subedi et al., 2018	

Those mutations/polymorphisms that are previously described are underlined. del(deletion). *Mutations previously detected associated with increased antimicrobial resistance.

*^a^FQs, fluoroquinolone; RIF, rifampicin; COL, colistin; IMI, imipenem; MER, meropenem; CEF, cefepime; AMGs, AMGnoglycosides; CAZ/AVI, ceftazidime/avibactam; C/T, ceftolozane/tazobactam; PPT, piperacillin-tazobactam.*

### Diversity of Structures Surrounding *bla*_VIM–__2_ in *Pseudomonas aeruginosa* Isolates

**FIGURE 2:**
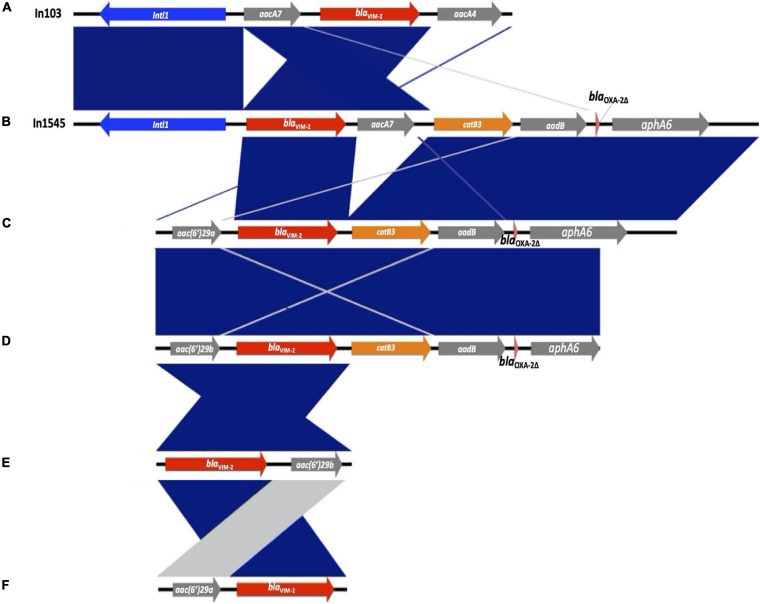
Genetic comparison of the *bla*_VIM–__2_-containing class 1 integrons detected in this study and gene cassette arrays. The different types of gene cassette arrays identified are designated from **(A)** to **(F)**. Genes are denoted by horizontal arrows with their corresponding transcription orientations. *bla*_VIM–__2_ is shown with red arrows. Figure was created using EasyFig.

The *bla*_VIM–__2_ gene was associated with six different types of gene cassette arrays encoding resistance to aminoglycosides or chloramphenicol, designated from A to F (see Figure 2). The type E was found in three isolates ST111, the type B in two isolates ST357 and ST1027, and the types A, C, D, and F were found in one isolate each, with different STs (type A—ST1249; types C, D, and F ST111; see Figure 1). We found two of these *bla*_VIM–__2_ gene cassette arrays within class 1 integrons; the In103 (type A) was first reported in one isolate of *P. aeruginosa* from Portugal in 2018 (accession number AY954726; Botelho et al., 2018) and a new integron designated as In1545 (type B) including the *bla*_VIM–__2_-*aacA7*-*catB3*-*aadB*-*bla*_OXA–__2__Δ_:ISAbA125:*aphA6* gene cassette. In addition, two isolates recovered from the same patient showed a similar gene cassette to In1545, but differ by the lack of the *aacA7* and presence of the upstream region of *bla*_VIM–__2_ of *aac(6′)29a* (type C) in one isolate and *aac(6′)29b* (type D) in the other isolate. Furthermore, we found two cassette arrays (types E and F) similar to a region of In59 previously reported in Colombia (Correa et al., 2015; see Figure 2).

Interestingly, in six isolates, the class 1 integrons were associated with the other gene cassette; the In1237 was detected in one isolate from hospital 2 (C2-42), containing the gene cassette *qnrvc*-*gcu165*-*arr2*-*dfrA22e*, which confers resistance to fluoroquinolones, rifampicin, and trimethoprim. This integron was previously identified in *P. aeruginosa* in France in 2016 (accession number KU984332; Janvier et al., 2017). Also, a new integron designated as In2011 was detected in five isolates (C1-133-2, C2-64-3, C2-64-2, C2-30-1, and C2-42-1) from hospitals 1 and 2, containing the gene cassette *gcu183*-*aacA4′*-*aadA1*_Δ_*32* -*bla*_OXA–__10_, which confers resistance to aminoglycosides and β-lactam. This integron carries a gene cassette *gcu183* without a recognized function yet (Liapis et al., 2019). Additionally, the gene cassette structures of In1237 and In2011 were detected in three (C2-42-1, C2-155-2, and C2-101) and one (C2-42) isolates from hospital 2, respectively.

The sequence analysis of class 1 integrons with both gene cassette arrays and promotor showed that In103 contained strong Pc variant PcS, which contributes significantly to the expression of gene cassettes and the IntI1 variant IntI1 _R–__32___*H*__39_, while In1545 and In2011 contain the weak Pc variant PcH1 and the IntI1 variant IntI1_R–__32___*H*__39_, which has been associated with an efficient excision activity (Jové et al., 2010). However, it has been suggested that this ability might compensate for the weak expression of antibiotic resistance and could enhance the capacity of the integron to adapt to antibiotic pressure and thus represent a survival advantage (Vinué et al., 2011).

### Transferring a Plasmid Carrying *bla*_KPC–__2_ in a Case of Infection by *Pseudomonas aeruginosa*

**FIGURE 3:**
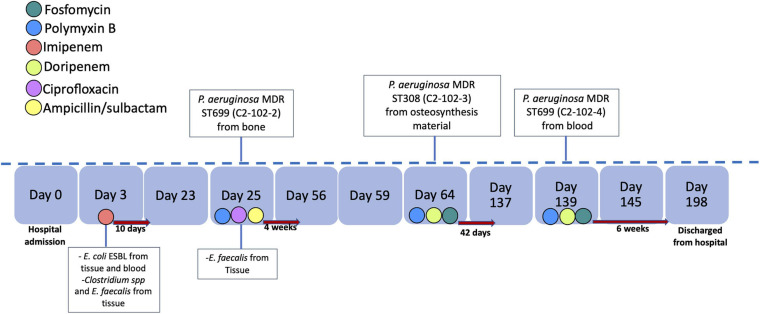
Timeline of antimicrobial treatment and bacterial identification. The antibiotics are indicated by color and the time of treatment with a red arrow to the right. We indicate the bacteria and source of isolation in the day of identification.

The *bla*_KPC–__2_ gene was not detected in the genome in five of the seven isolates of *P. aeruginosa* with positive PCR suggesting the loss of the plasmid or the *bla*_KPC–__2_ gene during storage or subculture processing prior to sequencing. However, in two of the three isolates recovered from the same patient, the *bla*_KPC–__2_ gene was detected in the genome. The strains were isolated from a patient in hospital 2 with chronic pelvic osteomyelitis with the first strain an MDR *P. aeruginosa* (ST699/C2-102-2 strain). Subsequently, osteomyelitis treatment failed due to the retention of the osteosynthesis material, with a second isolate of MDR *P. aeruginosa* (ST308/C2-102-3 strain) from the osteosynthesis material. A third relapse of the infectious occurred, and an MDR *P. aeruginosa* (ST699/C2-102-4 strain) was isolated in blood cultures. The timeline of the antimicrobial treatment and bacterial isolates of this case are shown in Figure 3.

**FIGURE 4:**
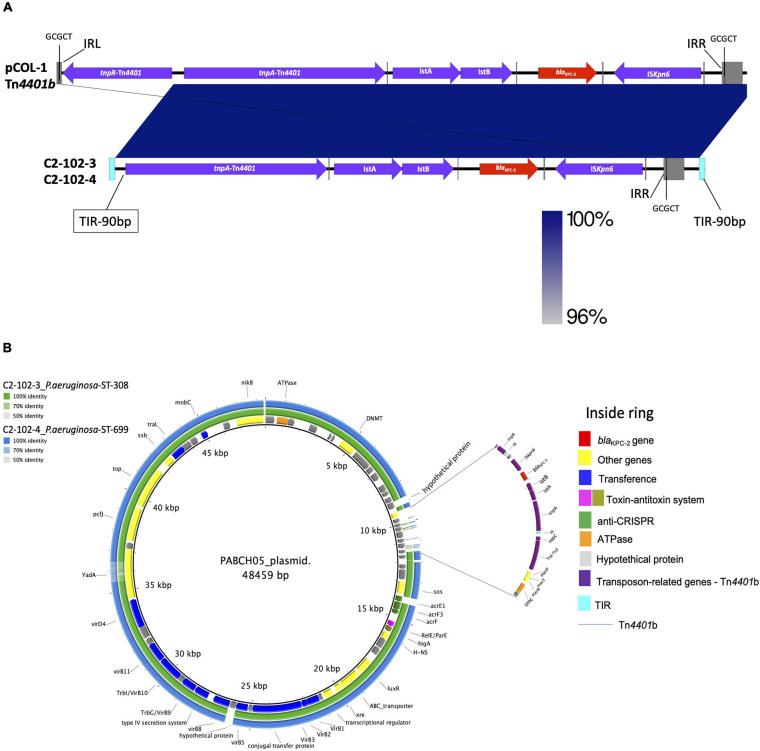
**(A)** Comparison of Tn*4401*b containing *bla*_KPC–__2_ gene identified in two isolates of *P. aeruginosa* from the same patient (C2-102-3 and C2-102-4) with isoform Tn*4401*b from *P. aeruginosa* COL-1 from Colombia (accession number KC609323.1) (18). Gray squares represent left and right inverted repeats (IRL and IRR, respectively) delimiting Tn4401b, flanked by GCGCT target site duplications (TSDs). Genes are denoted by horizontal arrows with their corresponding transcription orientations. Insertion sequences: Isk*pn6* and Isk*pn7* with IRL and IRR sequences (gray line); transposase *tnpA* and resolvase, *tnpR*. **(B)** BLAST Ring Image Generator (BRIG 0.95 and BLASTN v2.2.29) of comparison of the annotated plasmid from *P. aeruginosa* PABCH05 strain, Boston, MA, United States (accession number CP056099.1), with C2-102-3 and C2-102-4 isolates sequenced in this study. The internal ring shows the resistance and structural genes of the plasmid of the PABCH05 strain indicated by different colors (right panel). Green ring and blue ring correspond to the BLASTn result of C2-102-3 and C2-102-4 contigs relative to the plasmid reference (inside ring), respectively. The Tn*4401*b without the resolvase (tnpR) gene and surrounding is indicated by the blue lines. The transposon is flanked at both ends by terminal inverted repeats (TIRs) of 90 bp followed downstream by *tnpR* and upstream by *vapC*-*tnpA*-*merP*-*merT*-*merR* genes.

The isolates recovered from the osteosynthesis material (C2-102-3) and blood (C2-102-4) belonging to ST308 and ST699 carried *crpP*, *fosA*, and *catb7* conferring, respectively, quinolone, fosfomycin, and chloramphenicol resistance. Additionally, the C2-102-3 isolate carried *tetC*, *sul1*, *aph(3″)-Ib*, *aac(6′)Ib-cr*, *ant(2″)Ia*, *aph(6)-Id*, and *aac(6)-Ib3* conferring tetracyclin, sulfonamide, and aminoglycoside resistance, respectively (see Figure 1). Also, a new integron was detected and designated as In2012 including *aadB* (*ant(2″)-Ia*) and *aacA4′* (*aac(6)-Ib3*) gene cassettes, and containing the variants PcH1 and IntI1_R–__32___*H*__39_. Despite the different genetic backgrounds of C2-102-3 and C2-102-4 isolates described above, both isolates carried *bla*_KPC–__2_ on a transposon similar to Tn*4401*b identified previously in the pCOL1 plasmid from *P. aeruginosa* COL-1 in Colombia (accession number KC609323.1; Naas et al., 2013), without the resolvase gene (*tnpR*). Therefore, the GCGCT target site duplication (TSD) only was detected downstream to the IS*Kpn6* gene (see Figure 4A). Additionally, this ΔTn*4401*b was flanked at both ends by terminal inverted repeats (TIRs) of 90 bp followed downstream by *tpnR* and upstream by *vapC*-*tnpA*-*merP*-*merT*-*merR* genes.

Interestingly, a BLAST analysis revealed that 2 and 3 contigs obtained by *de novo* assembly of the C2-102-3 and C2-102-4 isolates, respectively, showed similarities to the backbone of a plasmid from the *P. aeruginosa* PABCH05 strain recovered in Boston, MA, United States (accession number CP056099.1), albeit only the region in the contigs that contained ΔTn*4401*b and the surrounding not matched with the plasmid (see Figure 4B). These findings suggest a novel plasmid likely generated through transposition and homologous recombination events. Our finding supports the notion that the *bla*_KPC–__2_ gene could have been horizontally transferred by this plasmid between different strains of *P. aeruginosa* in the same patient during the time of infection.

## Discussion

This study provides new data supporting the genetic diversity and differences in the genetic context of *bla*_VIM–__2_ and *bla*_KPC–__2_ of MDR *P. aeruginosa* isolates recovered from colonized and infected patients from two tertiary care hospitals, one in Medellín and the second located in a municipality close to Medellín, both areas with high carbapenemase endemicity in Colombia. We identified a remarkable variety of genetic backgrounds of *P. aeruginosa* isolates carrying *bla*_KPC__2_ or *bla*_VIM__2_, diversity of the genetic environment of *bla*_VIM_, as well as a possible event of spread of *bla*_KPC–__2_ mediated by a plasmid associated with a structure that contained part of Tn*4401*b in isolates from an infected case. This molecular heterogeneity suggests the potential of these resistant determinants to disseminate mediated by different MGE in *P. aeruginosa* in Colombia.

According to previous studies in Colombia, *bla*_VIM_ and *bla*_KPC_ have been the most frequent carbapenemase genes detected in *P. aeruginosa* and are widely disseminated in the country (Pacheco et al., 2014; Saavedra et al., 2014; Vanegas et al., 2014; Correa et al., 2015). In this study, we detected equal frequency of isolates of CRPA carrying *bla*_VIM–__2_ and *bla*_KPC–__2_ (*n* = 11, 47.8% for each), in contrast to other countries where *bla*_VIM–__2_ is widely spread (Hong et al., 2015). We detected that most isolates carrying *bla*_VIM–__2_ were recovered from colonized patients, while *bla*_KPC–__2_ was mostly from infected patients. Furthermore, we found one isolate co-harboring *bla*_KPC–__2_ and *bla*_VIM–__2_ from a patient with urinary tract colonization, which was previously described in infected patients (Correa et al., 2012, 2015; Vanegas et al., 2014; Pacheco et al., 2019). Studies of molecular characterization in other countries and in Colombia previously focused mainly on infections caused by CPPA (Correa et al., 2015; Vanegas et al., 2014), in contrast to our study where we found colonization with CRPA harboring VIM and KPC, which is a major infection control concern (Abdalhamid et al., 2016). Interestingly, all the patients who were colonized by CRPA had a record of previous hospitalization, and most of them were referred from other hospital localized in different municipalities near Medellin and more than half had previous antibiotic exposure (see Supplementary Table 1). Antimicrobial pressure is a risk factor associated with the colonization of XDR *P. aeruginosa* in previous studies (Buhl et al., 2015).

Different ST profiles (*n* = 5) were identified among the isolates harboring *bla*_KPC–__2_ analyzed by both rep-PCR/diversilab and WGS (*n* = 7). ST309 is a potential high-risk clone reported in the isolates of *P. aeruginosa* from Mexico, carrying *bla*_KPC–__2_ and important virulence factors involved in colonization and dissemination, also described in two extensively drug-resistant isolates from US carrying *bla*_GES–__19_ and *bla*_GES–__26_ (Morales-Espinosa et al., 2017; Khan et al., 2019). ST308, a clone associated with higher virulence, was reported before in Colombia and other countries of South America, Europe, Asia, and Oceania (Cuzon et al., 2011; Del Barrio-Tofiño et al., 2020). The isolates belonging to ST309 and ST308 were associated with the serotype O11, documented in several high-risk clones also (Del Barrio-Tofiño et al., 2020). Other isolates belonging to ST313 and ST699 were associated with other serotypes, all previously described from different continents, widely disseminated (Libisch et al., 2008; Ji et al., 2013; Cholley et al., 2014). Likewise, among the isolates harboring *bla*_VIM–__2_ (*n* = 9), various ST profiles (*n* = 4) were identified, with a main linage ST111 associated with the O12 serotype, the second more widespread high-risk clone after ST235, which disseminated in different Colombian cities and has been reported in other countries of America, Europe, and Asia (Vanegas et al., 2014; Correa et al., 2015; Del Barrio-Tofiño et al., 2020). Interestingly, isolates that belonged to ST111 from rectal swabs and sites of infection from two patients from hospital 2 were genetically related. Other isolates belonged to ST1249 described previously (Vanegas et al., 2014), and ST357 and ST1027 reported in other countries (Hrabák et al., 2011; Horna et al., 2019; Nawfal Dagher et al., 2019; Khan et al., 2020). These findings reflect a variety of genetic backgrounds of MDR *P. aeruginosa* isolates carrying *bla*_KPC__2_ or *bla*_VIM__2_ due to the dissemination of successful international clones and the emergence of other clones in this area of Colombia, associated to the widespread dissemination mediated by MGEs.

Our analysis of WGS revealed that *bla*_VIM–__2_ was associated with different gene cassette arrays encoding resistance to other antibiotics such as aminoglycosides and chloramphenicol, among isolates with different ST (see Figure 2). Some isolates carried *bla*_VIM–__2_-containing class 1 integrons including In103 (ST1249) and a new integron designated In1545 (ST357 and ST1027) whose cassette genes were detected in two isolates ST111 from the same patient but differ by the lack of *aacA7*, as well as a different upstream region of *bla*_VIM–__2_ for each isolate (see Figure 2), suggesting gene cassette rearrangement. Previous studies demonstrated that under antimicrobial pressure, the IntI-mediated rearrangement can generate integron variants (Barraud and Ploy, 2015). Additionally, we found coexistence with infrequent or new integrons with other gene cassettes that confer resistance to fluoroquinolones, rifampicin, trimethoprim, and β-lactam (In2011 and In1237). This is consistent with previous studies from other countries that showed a high prevalence of class 1 integrons with a high diversity of gene cassettes among MDR *P. aeruginosa* isolates (Botelho et al., 2018). Likewise, the detection of some mutational mechanisms of resistance showed the propensity to develop the MDR phenotype in the isolates of the study.

Another important finding in our study are the isolates recovered at different times from the same patient that showed heterogeneous genetic backgrounds but the same location of the *bla*_KPC–__2_ gene within a transposon similar to Tn*4401*b of the pCOL1 plasmid (Naas et al., 2013), without the resolvase (encoded by gene *tnpR*). Supported by the clinical data and BLAST analysis, we hypothesized that the *bla*_KPC–__2_ gene could have been horizontally transferred by one plasmid that carried the transposon between the different strains of *P. aeruginosa* in the same patient during the infection period (see Figure 4B). There are a few reports of *bla*_KPC_ inter- and intraspecies transfer within patients (Goren et al., 2010; Adler et al., 2016), but some studies have demonstrated *in vivo* acquisition of an insertion sequence or plasmid harboring *bla*_KPC–__2_ among *Enterobacterales* (Ding et al., 2016). The acquisition also suggests that under antimicrobial pressure, the transposition of insertion sequences or the movement of plasmid among coinfected strains may emerge. In our case, these events could have occurred because the patient had broad-spectrum antimicrobial therapy and several infection relapses secondary to the osteosynthesis material. Future long read sequencing studies are required to confirm the complete sequence of this plasmid.

Overall, in this study, most of the patients colonized and infected by CRPA were older adults (>63 years old), with different underlying conditions, with various medical devices and broad antibiotic exposure, mainly to carbapenems, piperacillin-tazobactam, and glycopeptides (see Supplementary Table 1). Exposure to broad-spectrum antibiotics has been described as the main factor related to carbapenems resistance (Richter et al., 2019). Furthermore, a multidrug resistance phenotype was detected in more than half of CPPA and non-CPPA isolates, a phenomenon locally described only in CPPA (Vanegas et al., 2014). These differences might be explained by the inclusion of isolates recovered from rectal swabs in colonized patients in this study, giving the possibility of acquisition of different resistance genes in these isolates because the gastrointestinal tract is the main source of resistant *Enterobacterales* and can play a key role in the spread of antibiotic resistance by horizontal transmission (Abdalhamid et al., 2016).

Some limitations of this study include that only isolates from two hospitals were analyzed, and most of these were collected from a single institution, limiting the extrapolation of the results. On the other hand, five *P. aeruginosa* isolates could have lost the *bla*_KPC_ gene or the plasmid that contained it during storage or subculture processing prior to sequencing. Therefore, it was not possible to define the genetic environment of the *bla*_KPC_ gene in those isolates.

In conclusion, the dissemination of *bla*_VIM–__2_ and *bla*_KPC–__2_ in *P. aeruginosa* in this area in Colombia has been strongly influenced by successful international clones and emergence of new clones carrying these genes, as well as the presence of resistance determinants in integrons, transposable elements, and plasmids, accompanied by gene rearrangement likely through transposition and homologous recombination. We postulate that the antimicrobial pressure may have played an important role. Infection control strategies and rational antibiotic use may help limit the spread. In addition, surveillance of colonization patients may also limit the subsequent infection and dissemination of these bacteria.

## Data Availability Statement

The datasets presented in this study can be found in online repositories. The names of the repository/repositories and accession number(s) can be found in the article/Supplementary Material.

## Author Contributions

ED and AR made the significant contributions to laboratory processing. CC, CA, and CP made the collection and analysis of clinical data. AR performed the sequence data processing, analysis and wrote the manuscript, which was reviewed by all authors.

## Conflict of Interest

MV has received grant support from the Merck Inc., Pfizer, and WestQuímica. The remaining authors declare that the research was conducted in the absence of any commercial or financial relationships that could be construed as a potential conflict of interest.

## Publisher’s Note

All claims expressed in this article are solely those of the authors and do not necessarily represent those of their affiliated organizations, or those of the publisher, the editors and the reviewers. Any product that may be evaluated in this article, or claim that may be made by its manufacturer, is not guaranteed or endorsed by the publisher.

## Footnotes

1
http://rast.nmpdr.org

2
https://cge.cbs.dtu.dk/services/MLST/

3
https://cge.cbs.dtu.dk/services/ResFinder/

4
https://cge.cbs.dtu.dk/services/PAst/

5
http://integrall.bio.ua.pt

6
http://mjsull.github.io/Easyfig/
