# Supplementary material for: Genetic Diversity of Multidrug-Resistant Pseudomonas aeruginosa Isolates Carrying blaVIM–2 and blaKPC–2 Genes That Spread on Different Genetic Environment in Colombia
Source: Front Microbiol. 2021 Aug 27;12:663020. doi: 10.3389/fmicb.2021.663020 (PMC8432936; doi:10.3389/fmicb.2021.663020)
Supplement: Supplementary file 2 [file Data_Sheet_2.PDF]

Supplementary Material

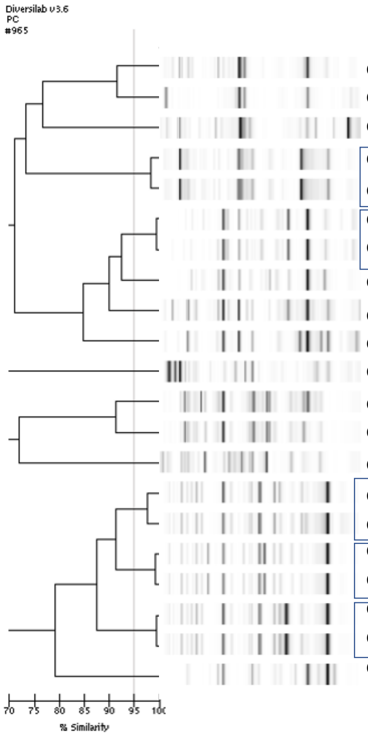

Isolates	Year of isolation	Source	Ward	Variant of *bla* KPC	Variant of *bla* VIM	WGS	
C2-100-1	2014	Bone	Non-ICU	***bla*** $_{KPC-2}$	–	✓	
C1-67	2014	Urine	ICU	–	***bla*** $_{VIM-2}$	✓	
C2-102-2*	2014	Bone	Non-ICU	***bla*** $_{KPC-2}$	–	✓	
C2-53-3	2014	Rectal swab	Non-ICU	***bla*** $_{KPC-2}$	–		1
C2-54	2014	Tissue	Surgery	***bla*** $_{KPC-2}$	–	✓	1
C2-105-1	2014	Tissue	Non-ICU	***bla*** $_{KPC-2}$	–	✓	2
C2-102-3*	2015	Osteosynthesis material	Surgery	***bla*** $_{KPC-2}$	–	✓	2
C2-144	2015	Peritoneal fluid	Non-ICU	***bla*** $_{KPC-2}$	–	✓	
C2-155-2	2015	Rectal swab	ICU	–	***bla*** $_{VIM-2}$	✓	
C2-146-2	2015	Rectal swab	Non-ICU	–	***bla*** $_{VIM-2}$		
C2-85-5	2014	Blood	Non-ICU	***bla*** $_{KPC-2}$	–		
C2-11	2014	Rectal swab	Non-ICU	***bla*** $_{KPC-2}$	–		
C2-12	2014	Blood	Non-ICU	***bla*** $_{KPC-2}$	–		
C2-101	2014	Rectal swap	Non-ICU	–	***bla*** $_{VIM-2}$	✓	
C1-133-2	2014	Tracheal aspirate	ICU	–	***bla*** $_{VIM-2}$	✓	3
C2-30-1	2014	Bone	Non-ICU	–	***bla*** $_{VIM-2}$	✓	3
C2-42*	2014	Rectal swab	Non-ICU	–	***bla*** $_{VIM-2}$	✓	4
C2-42-1*	2014	Secretion	Surgery	–	***bla*** $_{VIM-2}$	✓	4
C2-64-2*	2014	Tissue	Non-ICU	–	***bla*** $_{VIM-2}$	✓	5
C2-64-3*	2014	Rectal swab	Non-ICU	–	***bla*** $_{VIM-2}$	✓	5
C2-102-4*	2015	Blood	Non-ICU	***bla*** $_{KPC-2}$		✓	

* Isolates from the same patient with the same color

Figure S1. Dendrogram depicting genetic relatedness of carbapenemases-producing *P. aeruginosa* isolates by repetitive sequence-based PCR (rep-PCR). We highlight carbapenemases genes *bla*$_{KPC-2}$ or *bla*$_{VIM-2}$ identified in each isolate, and who were selected to whole genome sequencing. Boxes indicate the five rep-PCR types (1-5) found.

Table S1 Demographic and clinical characteristics of patients infected or colonized by Carbapenem-resistant *Pseudomonas aeruginosa* n=41

**Variable**	**Overall (n=41) n(%)**	**Infected (n=24) n(%)**	**Colonized (n=17) n(%)**	**p-value**
Age, median (IQR)	63 (49-74)	57(44.5-72)	66(54.5-76.5)	0.2
Gender				
Male	28(68.3)	16(66.6)	12 (70.6)	1.0
Days of hospital stay before sampling, median (IQR)	4 (2-24)	13.5(2.5-27.5)	2(1.5-13.5)	0.15
History in past 6 month				
Hospitalization	34(82.9)	17(70.8)	17(100)	0.03
Stay in ICU	10(24.4)	4(16.7)	6(35.3)	0.27
Dialysis	4(9.8)	1(4.2)	3(17.6)	1.0
Hospital location				
AICU	7(17.1)	4(16.7)	3(17.6)	1.0
Adult wards	34(82.9)	20(83.3)	14(82.4)	1.0
Medical devices				
Urinary catheter	8(19.5)	6(25)	2(11.8)	0.43
Central venous cateter	6(14.6)	3(12.5)	3(17.6)	0.68
Invasive Mechanical Ventilation	6(14.6)	4(16.7)	2(11.8)	1.0
Endotracheal tube	1(2.4)	0(0.0)	1(3.7)	0.42
Gastric tube	6(14.6)	5(20.8)	1(3.7)	0.37
Isolation sample				
Rectal swab	12(29.3)	0(0)	12(70.6)	
Soft tissue	7(17.1)	7(29.2)	0(0)	
Respiratory sample[a]	3(4.9)	2(8.3)	1(5.9)	
Urine	5(12.2)	3(12.5)	2(11.8)	
Bone	6(14.6)	4(16.6)	2(11.8)	
Blood	5(12.2)	5(20.8)	0(0)	
Sterile body fluids[b]	4(9.8)	4(16.6)	0(0)	
Comorbidities				
Diabetes	10(24.4)	5(20.8)	5(29.4)	0.71
COPD	4(9.8)	2(8.3)	2(11.8)	1.0
CKD	6(14.6)	3(12.5)	3(17.6)	0.68
Coronary heart disease	6(14.6)	3(12.5)	3(17.6)	0.68
Hypertension	20(48.8)	11(45.8)	9(52.9)	0.76
Antibiotic exposure				
Preceding antibiotic exposure	19(46.3)	10(41.7)	9(52.9)	0.69
Carbapenems	5(12.2)	3(12.5)	2(11.8)	1.0
Fluoroquinolones	4(9.8)	2(8.3)	2(11.8)	1.0
Piperacilin-tazobactam	5(12.2)	3(12.5)	2(11.8)	1.0
Aminoglycoside	4(9.8)	2(8.3)	2(11.8)	1.0
Glycopeptides	5(12.2)	2(8.3)	3(17.6)	1.0

IQR, interquartile range.

AICU, Adult Intensive Care Unit

AITU, Adult Intensive Therapy Unit

COPD, Chronic Obstructive Pulmonary Disease

CKD, Chronic kidney disease

[a]Respiratory sample: Sputum, tracheal aspirate and oropharyngeal swab

[b]Sterile body fluids: Pleural fluid and peritoneal fluid

Table S2 Percentages of isolates resistance to antibiotics among carbapenemase-producing and non-carbapenemase-producing *P. aeruginosa* isolates.

**Antibiotic**	**(n=46) n(%)**	**CPPA (n=23) n(%)**	**non-CPPA (n=23) n(%)**	**p-value**
Piperacillin/Tazobactam (PIP-TZ)	34(73.9)	20(87)	14(60.9)	0.09
Amikacin (AK)	19(41.3)	11(47.8)	8(34.8)	0.55
Gentamicin (GM)	26(56.5)	17(73.9)	9(39.1)	0.04
Cefepime (CEF)	27(58.7)	17(73.9)	10(43.5)	0.07
Ceftazidime (CAZ)	26(56.5)	16(69.6)	11(47.8)	0.23
Imipenem (IMP)	39(84.8)	23(100)	20(86.9)	0.23
Meropenem (MER)	40(87)	22(95.7)	19(82.6)	0.35
Doripenem (DOR)	42(91.3)	23(100)	21(91.3)	0.49
Ciprofloxacine (CIP)	28(60.8)	15(65.2)	13(56.5)	0.76
>3 antimicrobial categories	34(73.9)	20(86.9)	15(65.2)	0.17
AK or GM + CEF + CAZ + IMI + MER + DOR +PIP-TAZO + CIP	15(32.6)	12(52.2)	3(13.0)	0.01
